# Interleukin-8 holds promise to serve as a molecular adjuvant in DNA vaccination model against *Streptococcus iniae* infection in fish

**DOI:** 10.18632/oncotarget.13728

**Published:** 2016-11-30

**Authors:** Erlong Wang, Bo Long, Kaiyu Wang, Jun Wang, Yang He, Xingli Wang, Qian Yang, Tao Liu, Defang Chen, Yi Geng, Xiaoli Huang, Ping Ouyang, Weimin Lai

**Affiliations:** ^1^ Department of Basic Veterinary, College of Veterinary Medicine, Sichuan Agricultural University, Chengdu, Sichuan, China; ^2^ Key Laboratory of Animal Disease and Human Health of Sichuan Province, Sichuan Agricultural University, Chengdu, Sichuan, China; ^3^ Department of Aquaculture, College of Animal Science and Technology, Sichuan Agricultural University, Chengdu, Sichuan, China

**Keywords:** aquaculture, bacterial diseases, DNA vaccine, interleukin-8, molecular adjuvant, Immunology and Microbiology Section, Immune response, Immunity

## Abstract

DNA vaccines had been widely used in animal models against various viral infections, while it was not so convincing for many infectious diseases especially bacterial disease in aquaculture. Interleukin-8(IL-8) as one of the CXC chemokines, its immunological role and adjuvant potential which had been proved in mammals were rarely reported in fish species. In this study, recombination plasmid pcDNA3.1/IL-8(pcIL-8) was conducted and the capacity of IL-8 as molecular adjuvant was explored from several aspects by co-injecting with a DNA vaccine encoding a-enolase(pcENO) against *Streptococcus iniae* infection in channel catfish. The results suggested that co-injection of pcIL-8 with DNA vaccine increased the innate immunity and specific antibody levels, as well as increased the immune-related genes involving in pro-inflammatory response, humoral and cellular immunity. Moreover, pcIL-8 enhanced the immunoprotection of pcENO with the relative percent survival(RPS) of 60% to 80% against S.in*iae* infection at 4 week post vaccination(p.v.), with the significantly higher RPS of 73.33% in pcENO+pcIL-8 group compared with that of pcENO alone(53.33%) at challenge test of 8 weeks p.v. Taken together, these results indicate pcIL-8 as a molecular adjuvant co-injected with DNA vaccine not only improves the immunoprotection but also maintains long period of immunity for channel catfish against S.in*iae* infection. Our study signifies that IL-8 holds promise to serve as a potential adjuvant in DNA vaccines against bacterial infections for long time.

## INTRODUCTION

Aquaculture as one of the most vibrant sector of the global food system has shown a rapid growth in the last decades and with such growth comes the need for effective control measures against increasingly diseases. Traditionally, controlling the disease and infection in aquaculture relies mainly on the use of antibiotics and antimicrobial compounds. However, several problems including antibiotic resistance and antibiotic residues have arisen due to the indiscriminate use of antibiotics which are not acceptable and even not permitted in some countries [1]. Therefore, seeking for the safe and efficacious vaccines is critical for a sustainable development of the aquaculture industry.

Compared with traditional inactivated and attenuated live vaccines, DNA vaccines consisting of naked plasmid DNA that will result in gene expression of immunoprotective antigen *in vivo* against fish diseases particularly viral pathogens, have made major advances over the last decade and become one of the most promising vaccine preparations in aquaculture [1]. Despite DNA vaccines used in fish have proven particularly efficacious against some viral diseases [2-7], only one DNA vaccine for aquaculture fish has been licensed for sale [8], and it is not so convincing for a number of other viral infections and bacterial disease. Scientists are now focused on improving and increasing the potency of DNA vaccines, which include the improvements of vectors and the co-vaccination of molecular adjuvants such as cytokines with the antigen to enhance the immune responses [9, 10].

Cytokines as a family of low molecular weight and secretory proteins are closely associated with the pro-inflammatory responses against invasive pathogens and the key regulators of the host immune system [11]. Several studies reported that the use of cytokines as vaccine adjuvants to augment the initial host responsiveness has been widely explored in mammals, and many cytokine genes like IL-1β and TNF-α have been identiﬁed in many fish species and many details concerning their immunological role are also demonstrated [11]. Chemokines, or chemotactic cytokines, are a family of cytokines involved in lymphocyte trafficking and immune surveillance within the immune system and served to attract leucocytes to particular sites [12]. Interleukin-8 (IL-8) as one of the first CXC chemokines to be discovered in fish has been cloned and characterized in few fish species [13-17], while the immunological role of IL-8 has not been achieved and details concerning its adjuvant potential are still lacking in these species.

Channel catfish (*Ictalurus punctatus*) as the most extensively cultured food-fish species has suffered from widespread disease outbreaks in recent years due to various bacterial pathogens such as *Streptococcus iniae*, a Gram-positive fish pathogenic bacterium with a broad host range [18-20]. Besides, channel catfish has been served as a classical model for the study of comparative immunology and research on the characterization and immunological role of its cytokines should help elucidate the immune system network in fish [17]. In this study, we further explore the adjuvant potential of IL-8 of channel catfish from several aspects by co-injecting with a DNA vaccine encoding α-enolase (pcENO) against *Streptococcus iniae* infection based on our previous report that IL-8 hold promise for use as potential immunopotentiator against bacterial infections but insufficient to extend the immune protection for long time in a subunit vaccine model [21].

## RESULTS

### Cloning, sequencing and eukaryotic expression plasmid of IL-8

**Figure 1:**
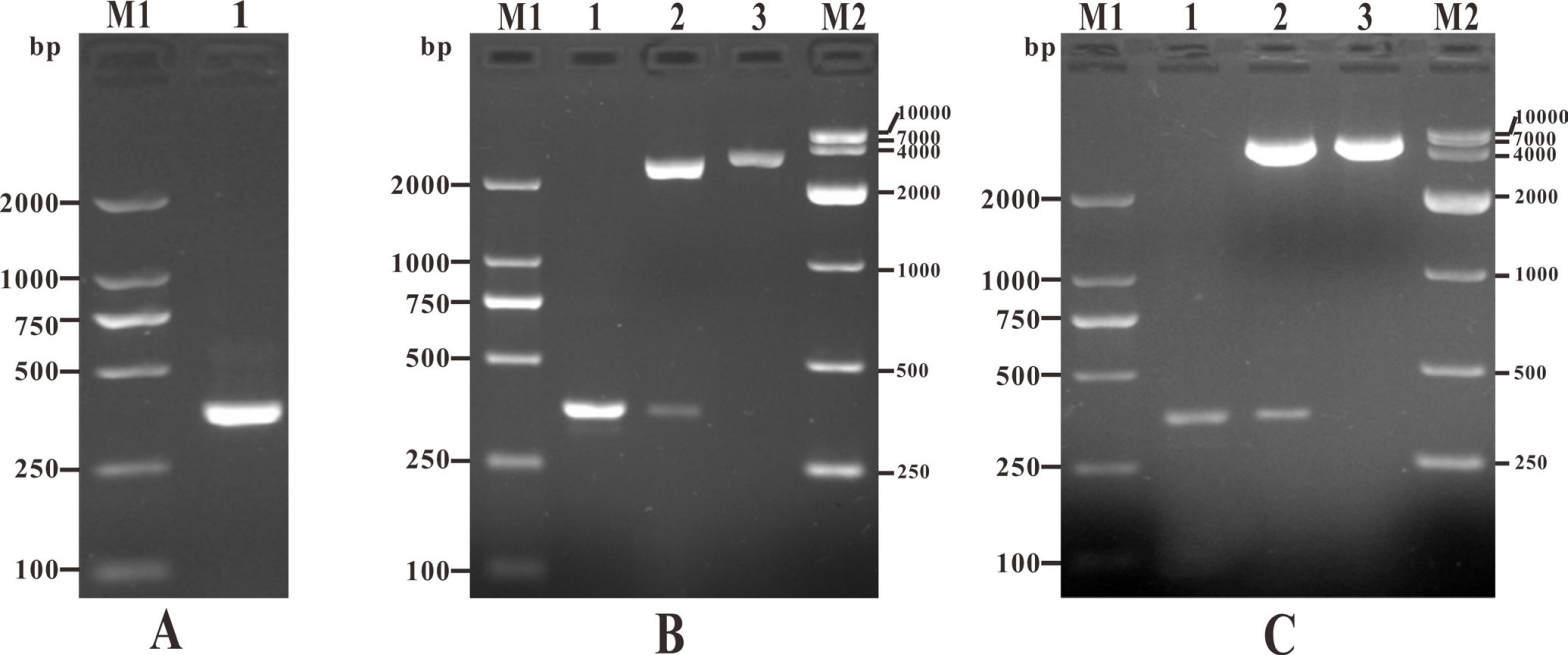
PCR amplification of IL-8 gene, detection of recombinant plasmid T-IL-8 and pcIL-8 **A**. PCR amplification of IL-8 gene. M1: DNA marker (DL2000); lane 1: PCR product of IL-8 gene (360bp). **B**. Identification of recombinant plasmid T-IL-8 with PCR and enzyme digestion. M1: DNA marker (DL2000); lane 1: PCR identification of T-IL-8; lane 2: digestion identification of T-IL-8 with Hind III and EcoR I; lane 3: digestion identification with EcoR I; M2: DNA marker (DL10000). **C**. Identification of recombinant plasmid pcIL-8 with PCR and enzyme digestion. M1: DNA marker (DL2000); lane 1: PCR identification of pcIL-8; lane 2: digestion identification of pcIL-8 with Hind III and EcoR I; lane 3: digestion identification with EcoR I; M2: DNA marker (DL10000).

**Figure 2:**
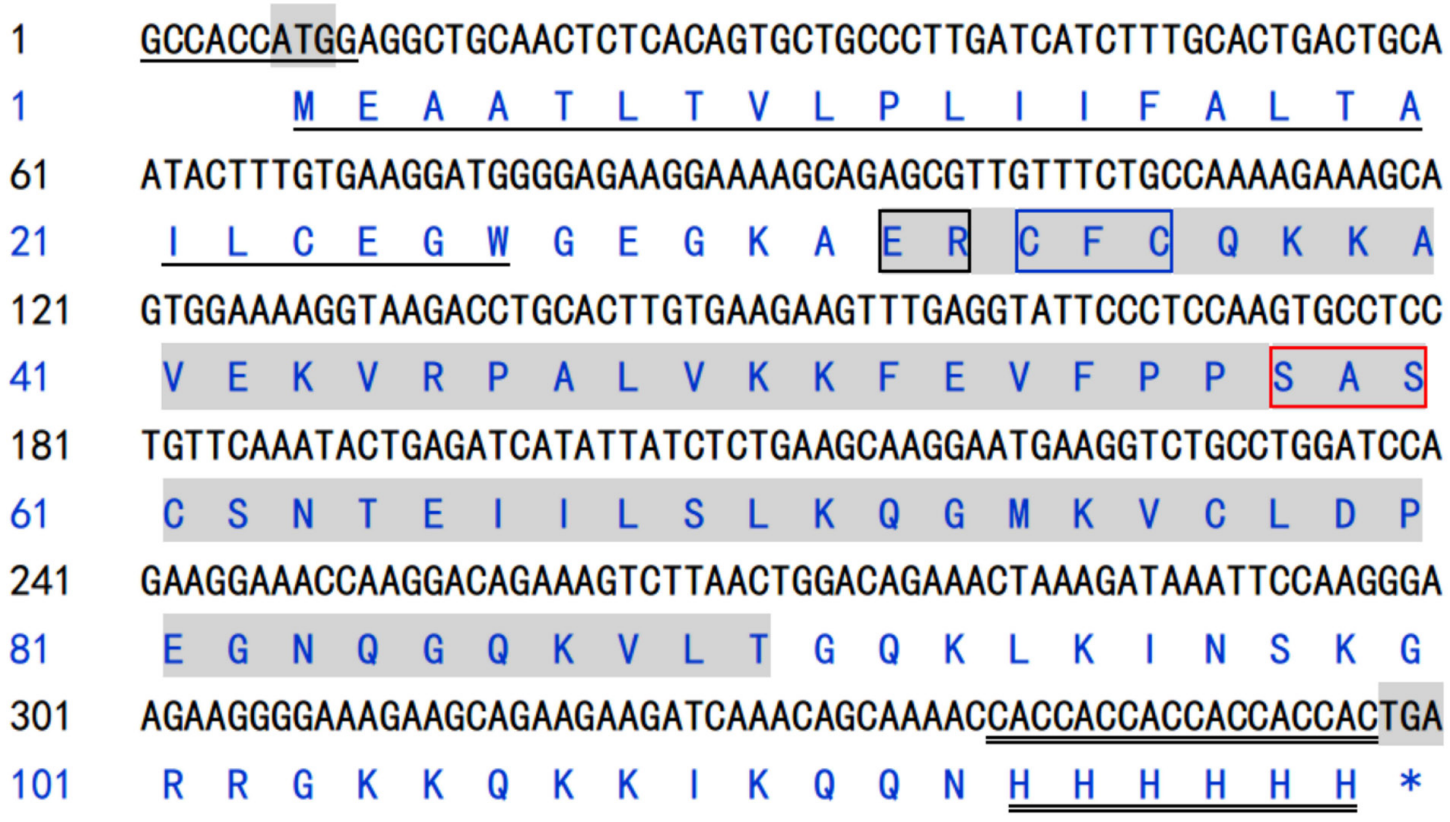
Nucleotide sequence and derived amino acid sequence of channel catfish IL-8 In the nucleotide sequence, underline presented a Kozak consensus sequence, double underline presented a 6×histidine tags sequence, the shadow presented initiation codon (ATG) and termination codon (TGA). In the amino acid sequence, underline indicated the predicted signal peptides, shadow regions indicated the conserved Chemokine_CXC domain, the ELR-like motif, CXC motif and GPH motif were indicated by black, blue and red box, respectively, double underline indicated the 6×histidine tags.

The channel catfish full length IL-8 gene was successfully PCR-amplified with 360 bp in length (Figure 1A), which contained a complete open reading frame (ORF) and a Kozak sequence at the N-terminus and 6×histidine tags sequence at the C-terminus. The IL-8 gene encode a 117 amino acid (a.a.) sequence with a predicted signal peptide (1~24 a.a.) and a conserved Chemokine_CXC domain belonging to Chemokine superfamily (30~88 a.a.). Moreover, three consensus motifs named as ELR-like motif which the leucine residue was not present, CXC motif and GPH motif were also found in the Chemokine_CXC domain of channel catfish IL-8 locating at the N-terminal of 30~31, 32~34 and 56~58 a.a. respectively (Figure 2). In addition, to construct the eukaryotic expression plasmid, IL-8 was successfully cloned into pMD19-T (Figure 1B) and inserted in vector pcDNA3.1 (Figure 1C).

### PCR detection of plasmid DNA in vaccinated fish

**Figure 3:**
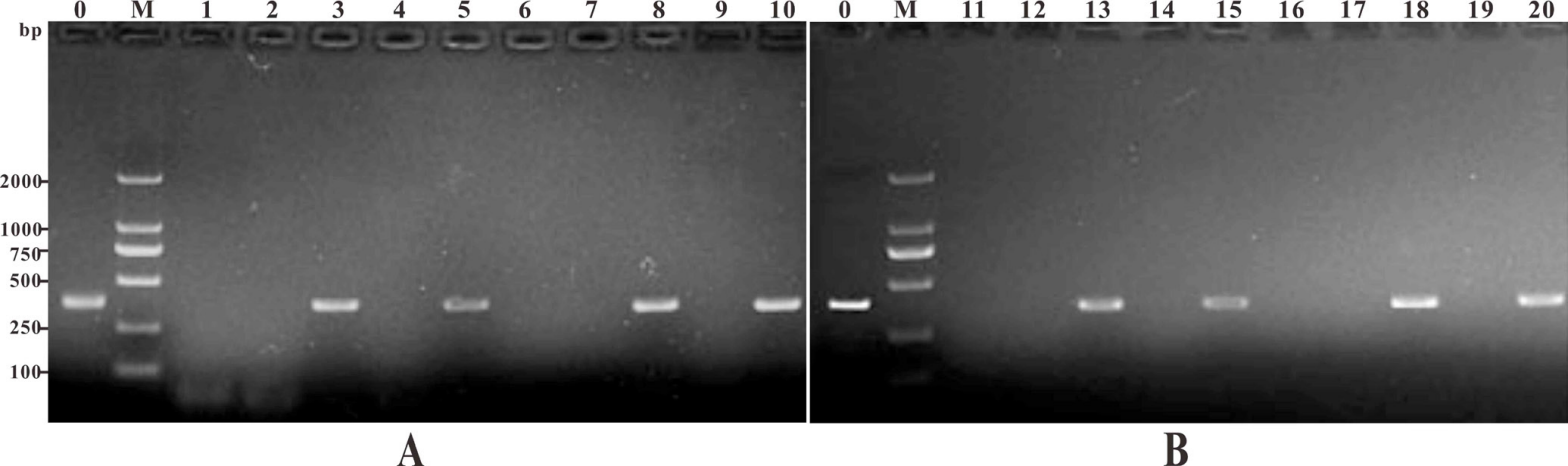
PCR detection of plasmid DNA in muscle of the vaccinated fish Channel fish were treated with PBS (lane 1, 6, 11, 16), or vaccinated with pcDNA3.1 (lane 2, 7, 12, 17), pcIL-8 (lane 3, 8, 13, 18), pcENO (lane 4, 9, 14, 19) and pcENO+pcIL-8 (lanes 5, 10, 15, 20) respectively. Muscle samples were taken and used for DNA extraction from the vaccinated fish at 7 days (lanes 1-5), 14 days (lanes 6-10), 28 days (lanes 11-15) and 56 days (lanes 16-20) p.v. respectively. Lane 0 (positive control): PCR product of plasmid pcIL-8, lane M: DNA markers (DL2000).

To confirm the presence of the pcIL-8 plasmid in muscle tissues at different time intervals, PCR was performed with specific primers. A PCR product in length of 360 bp was detected from DNA extracted from muscle tissues at 7, 14, 28 and 56 days p.v. in pcIL-8 group and pcENO+pcIL-8 group. No amplification was detected in DNA extracted from tissues of the PBS, pcDNA3.1 and pcENO group (Figure 3).

### Transcription analysis and expression of DNA vaccines

**Figure 4:**
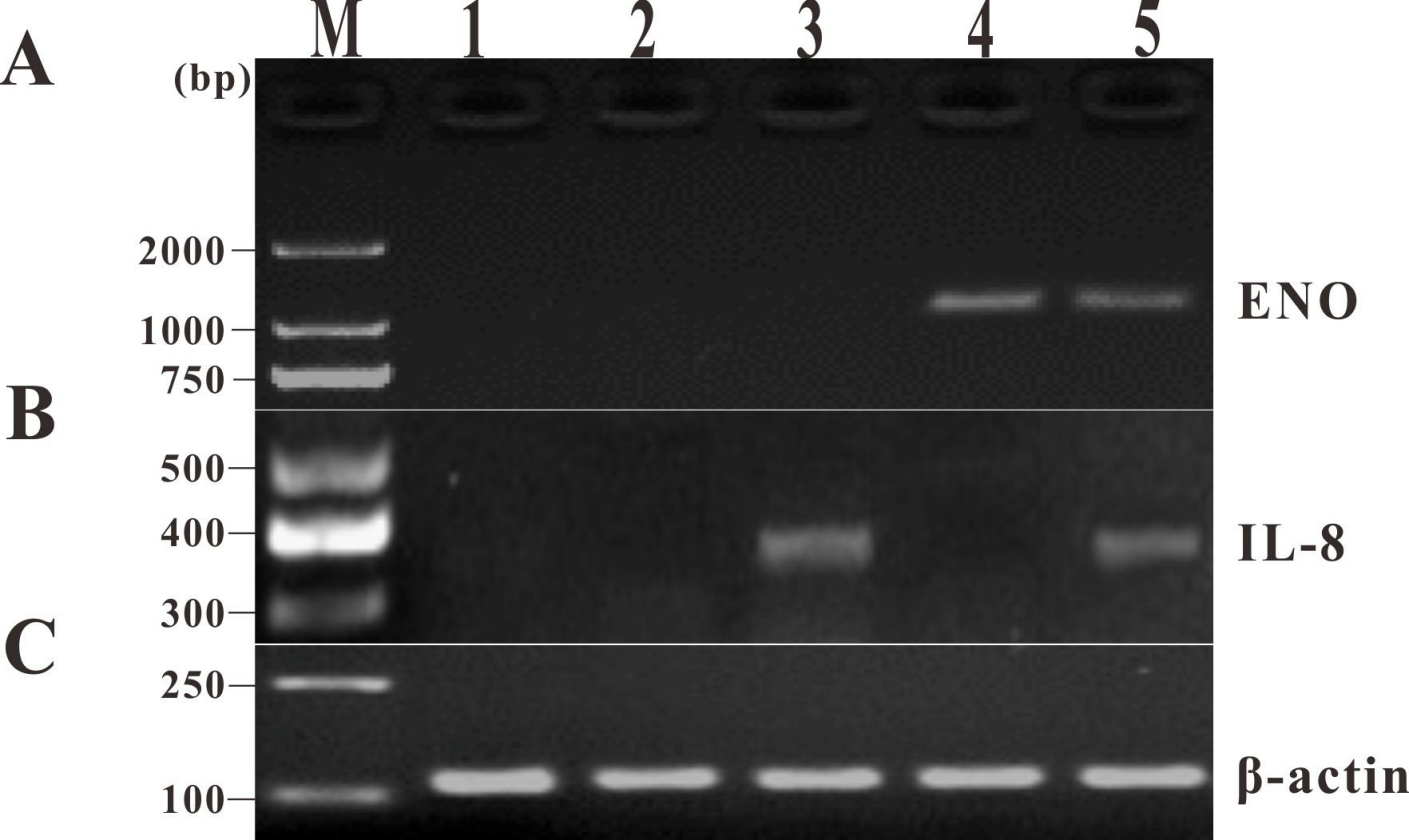
RT-PCR analysis of transcription of ENO gene and exogenous IL-8 gene in muscle tissues of the vaccinated fish at 14 days p.v **A**. ENO gene. **B**. Exogenous IL-8 gene. **C**. β-actin gene. M: DNA marker; lane 1~5, muscle injected with PBS, pcDNA3.1, pcIL-8, pcENO and pcENO+pcIL-8, respectively.

**Figure 5:**
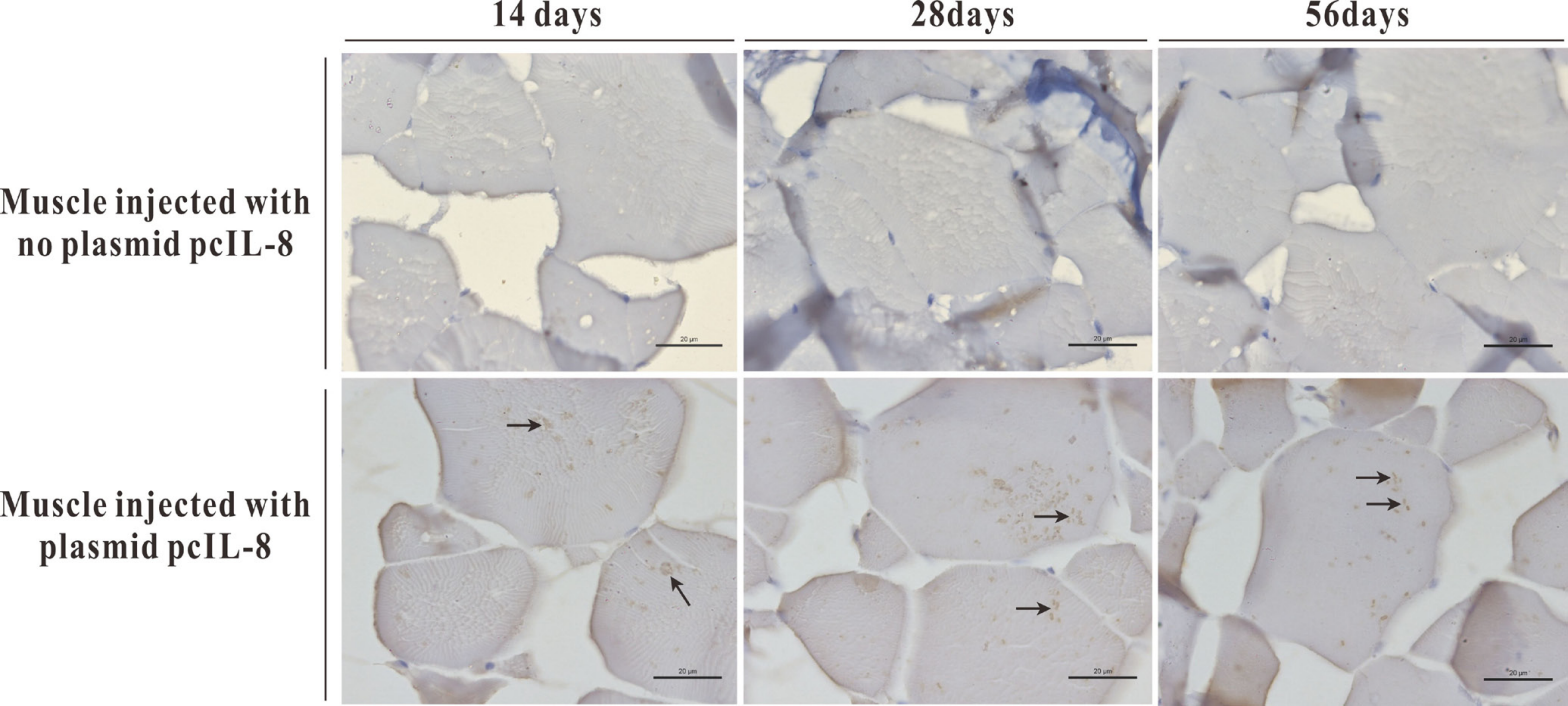
Immunohistochemistry analysis of exogenous IL-8 expressed in muscle tissues of the vaccinated fish at 14, 28 and 56 days p.v Bar = 20 μm.

Transcription analysis of ENO gene and exogenous IL-8 gene in muscle tissues of the vaccinated fish was performed by RT-PCR at 14 days p.v. The transcription of ENO gene was detected in the injected muscle of pcENO and pcENO+pcIL-8 group, but not in PBS, pcDNA3.1 and pcIL-8 group. Simultaneously, the transcription of exogenous IL-8 gene was detected in the injected muscle of pcIL-8 and pcENO+pcIL-8 group, not in PBS, pcDNA3.1 and pcENO group (Figure 4). Similarly, to examine whether exogenous IL-8 was expressed at translation level in the vaccinated fish, immunohistochemistry analysis was performed, which showed that the production of exogenous IL-8 was detected in muscle tissue of fish vaccinated with plasmid pcIL-8 groups (pcIL-8 and pcENO+pcIL-8 group) at 14, 28 and 56 days p.v., but not in that of fish vaccinated with no plasmid pcIL-8 groups (PBS, pcDNA3.1 and pcENO group) (Figure 5).

### Immunological analysis

**Figure 6:**
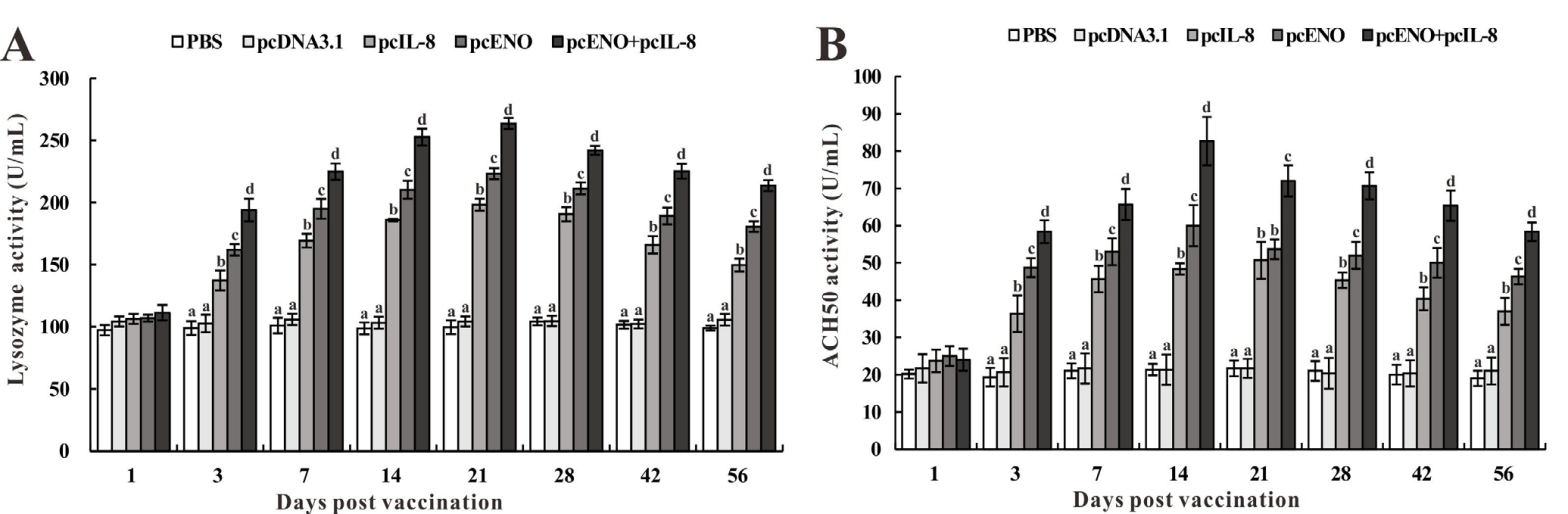
Serum lysozyme activity **A**. and ACH50 activity **B**. of vaccinated fish. Sera were collected at different time points p.v. from the fish vaccinated with PBS (control), pcDNA3.1, pcIL-8, pcENO and pcENO+pcIL-8. Data are presented as means ± SD (*n* = 5). Differences analysis between groups were determined using one-way ANOVA and Duncan's test. Different letters mean that the data of different groups in the same time differ significantly (*p* < 0.05). The same as below.

Serum lysozyme activity. The serum lysozyme activity of vaccinated fish was assessed by a turbidimetric assay at different time intervals post vaccination (Figure 6A). The results showed that the lysozyme activities of pcIL-8, pcENO and pcENO+pcIL-8 groups were significantly higher than that of PBS and pcDNA3.1 groups at all experimental time points except 1 day p.v. Moreover, pcENO and pcENO+pcIL-8 induced remarkably higher levels of lysozyme activity compared with those of pcIL-8 during the experiment. Although the difference of lysozyme activity in pcENO+pcIL-8 and pcENO was indistinctive at 1 day p.v., it was significant at other experimental time point. And the highest lysozyme activity were detected in pcENO+pcIL-8 group at 21 days p.v.

Serum ACH50 activity. During all experimental time points, the serum ACH50 activity in fish of pcIL-8, pcENO and pcENO+pcIL-8 groups were observably higher than that of PBS and pcDNA3.1 except 1 day p.v. and the difference between PBS and pcDNA3.1 group was indistinctive. Furthermore, pcENO and pcENO+pcIL-8 enhanced more markedly the action of ACH50 activity compared with pcIL-8 except 1 days and 21 days p.v. In addition, the ACH50 activity of fish vaccinated with pcENO+pcIL-8 was notably higher than that of pcENO except 1 day p.v., with the highest ACH50 activity occurring at 14 days p.v (Figure 6B).

### Specific serum antibody response

**Figure 7:**
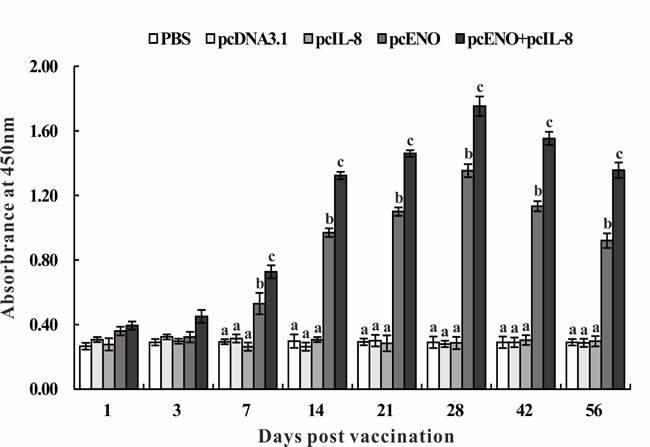
Specific serum antibody detection in vaccinated fish by ELISA Sera were collected at different days p.v. from the fish vaccinated with PBS (control), pcDNA3.1, pcIL-8, pcENO and pcENO+pcIL-8. Data are presented as means ± SD (*n* = 5). Different letters mean that the data of different groups in the same time differ significantly (*p* < 0.05).

The specific antibody in serum of vaccinated fish were assessed by ELISA at different days p.v. No specific anti-rENO antibody was detected in PBS, pcDNA3.1 and pcIL-8 groups during all the experimental time (Figure 7). Compared with above three groups, specific antibody was observed significantly (*p* < 0.05) in pcENO and pcENO+pcIL-8 groups from 7 to 56 days p.v. Moreover, pcENO+pcIL-8 stimulated the production of antibody significantly higher (*p* < 0.05) than pcENO at 7, 14, 21, 28, 42 and 56 days p.v. respectively, with the highest antibody level both peaking at 28 days p.v. (Figure 7).

### Expression of immune-related genes

**Figure 8:**
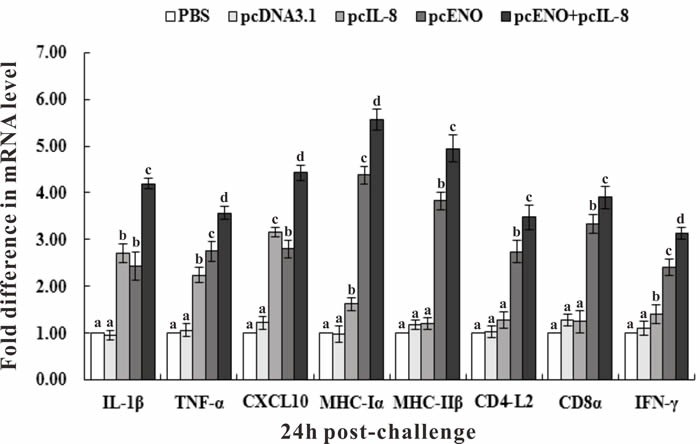
Expression of immune-related genes in vaccinated fish determined by qRT-PCR Channel catfish were vaccinated with PBS (control), pcDNA3.1, pcIL-8, pcENO and pcENO+pcIL-8. Total RNA was extracted from the head kidney at 24 h post-challenge of 4-week p.v. and used for qRT-PCR. For each gene, the mRNA level of the PBS-vaccinated fish was set as 1. Data are presented as means ± SD (*n* = 5). Different letters mean that the data of different groups in the same time differ significantly (*p* < 0.05).

qRT-PCR was performed to investigate the effect of vaccination on the expression of immune-related genes encoding interleukin 1β (IL-1β), tumor necrosis factor-α (TNF-α), CXC Chemokine Ligand 10 (CXCL10), major histocompatibility complex class Iα (MHC Iα) and IIβ (MHC IIβ), CD4-L2, CD8α and interferon-γ (IFN-γ) with β-actin (ACTB) as an internal control. The results showed that in fish of group pcIL-8, pcENO and pcENO+pcIL-8, the expression of all investigated genes were increased significantly compared with that in PBS and pcDNA3.1 groups except MHC IIβ, CD4-L2 and CD8α in pcIL-8 group, especially IL-1β, CXCL10, MHC Iα and MHC IIβ gene with more than 4-fold expression (Figure 8). Both pcENO and pcENO+pcIL-8 induced a significantly higher expression of TNF-α, MHC Iα, MHC IIβ, CD4-L2 and CD8α and IFN-γ compared with pcIL-8, while the expression of IL-1β and CXCL10 in pcENO group was lower than that of pcIL-8 group. Moreover, pcENO+pcIL-8 increased the expression of all investigated genes compared with pcENO alone.

### Immune protection against *S. iniae*

**Figure 9:**
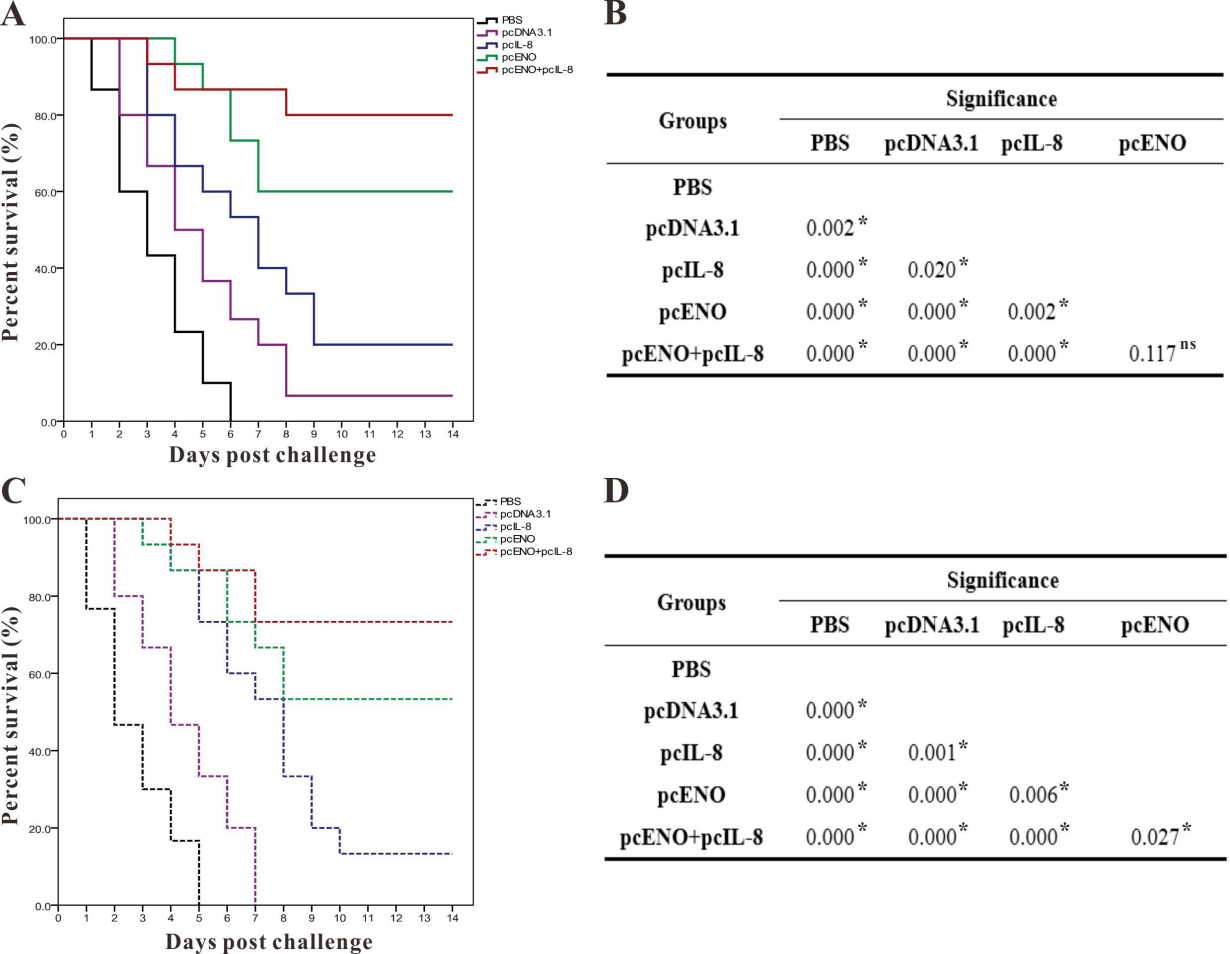
Percent survivals (Kaplan-Meier) of vaccinated fish (n = 30) during the challenges tests of 4 week p.v **A**. and 8 week p.v. **C**.. Differences of survivals among groups were tested using log-rank test shown in B (4 week p.v.) and D (8 week p.v.). The experiments were repeated twice independently with similar results. “*” denotes significant difference (*p* < 0.05), “ns” means not significant.

After the challenge experiment with the pathogenic S.in*iae* DGX07 at 4 weeks p.v, the accumulated survivals of fish vaccinated with PBS, pcDNA3.1, pcIL-8, pcENO and pcENO+pcIL-8 were 0%, 6.67%, 20.00%, 60.00% and 80.00% respectively (Figure 9A), which were equal to the value of corresponding PRS. Moreover, the survivals of group pcENO+pcIL-8 and pcENO were prominently higher (*p* < 0.05) than pcIL-8 group by log-rank test, while the difference of survivals in group pcENO+pcIL-8 and pcENO was not significant (Figure 9B).

To examine the duration of protection, fish were also challenged at 8-week p.v., and the results suggested that the cumulative survivals of fish vaccinated with PBS, pcDNA3.1, pcIL-8, pcENO and pcENO+pcIL-8 were 0%, 0%, 13.33%, 53.33% and 73.33% respectively (Figure 9C), which correspond to an RPS of 13.33% for pcIL-8 group, 53.33% for pcENO group and 73.33% for pcENO+pcIL-8 group respectively. In addition, the results of log-rank test showed that the survival in group pcENO+pcIL-8 and pcENO were remarkably higher (*p*< 0.05) than pcIL-8 group, which is consistent with the results of challenge at 4 weeks p.v, and the difference of survivals in group pcENO+pcIL-8 and pcENO was also significant (Figure 9D). Furthermore, S.in*iae* DGX07 was the only type of bacterial strain recovered from the liver, and kidney of moribund fish during two challenge tests, suggesting that mortality was caused by S.in*iae* DGX07 infection.

## DISCUSSION

During the past years, DNA vaccination has proven to be very effective in controlling some devastating viral diseases, but it is not so convincing for other viral infections and bacterial disease. Moreover, to enhance the immune responses and protective efficiency, increasingly focus is now to improve the plasmid vectors and co-vaccinate with molecular adjuvants such as cytokines in mammals. However, there is very little research related to the capacity of fish cytokines to serve as molecular adjuvants in DNA vaccination against viral and bacterial disease [9]. IL-8, as one of the first CXC chemokines discovered in fish, has been available and sequenced in some fish species, but its immunological role and adjuvant potential which have been proved in mammals [22, 23] are rarely reported in fish. In the present study, on the basis of our previous report, we have further explored the capacity of IL-8 as molecular adjuvant by co-injecting with a DNA vaccine against *S. iniae* infection in channel catfish.

Our previous results indicated that IL-8 hold promise for use as potential immunopotentiator in rENO subunit vaccine against *S. iniae* infections in channel catfish, but it is insufficient to extend the immune protection for long time [21]. In this study, we first constructed the recombination plasmid pcDNA3.1/IL-8 (pcIL-8), to enhance the transcription and translation efficiency and detect the expression of pcIL-8 *in vivo*, a Kozak consensus sequence (GCCACCATGG. A purine preferably A in position -3 and a G in position +4 have the strongest effects, modulating translation at least 10-fold) and a 6× histidine tag were insert into the primers for PCR amplication of IL-8. By the identification of gel electrophoresis and sequencing, the target sequence was 360 bp in length encoding a 117 amino acid sequence with a predicted signal peptide (1~24a.a.) and a conserved CXC Chemokine domain (30~88a.a.) containing three consensus motifs named as ELR-like motif, CXC motif and GPH motif respectively, which was different from other fish IL-8 lacking of ELR motifs [13, 14] and was belonging to the shorter alternatively spliced transcripts of IL-8 according to Chen et al [17].

Studies in mammals and teleosts have revealed that following intramuscular injection, DNA vaccines are subjected to several fates including localizing extracellularly at the injection site and being uptaken by local cells, degradation or transportation to other tissues [24, 25]. To determine whether the plasmid DNA was present and transcribed *in vivo*, DNA and RNA was extracted from the muscle tissues around the injection site for PCR and RT-PCR detection respectively. To detect the expression of pcIL-8 at translation levels in muscle of vaccinated fish, immunohistochemistry analysis was performed. The results showed that target genes (ENO gene and exogenous IL-8 gene) were detected in muscle tissue, suggesting that some plasmid DNA had escaped the fate of degradation and been uptaken by local cells, following by being expressed at transcription levels. Consistent with above results, the positive signals of exogenous IL-8 were detected in muscle of pcIL-8 and pcENO+pcIL-8 group by immunohistochemistry, which suggested exogenous IL-8 were expressed successfully in these groups, while no positive signals in muscle of other groups. These observations are similar to those reported previously in other fish species such as Japanese flounder [25, 26], rainbow trout [27], Atlantic cod [28] and Atlantic salmon [29].

Innate immunity as a fundamental defence mechanism plays an instructive role in the immune system and homeostasis in fish [30, 31]. Lysozyme activity and alternative complement activity (ACH_50_) are two important parameters to evaluate the host innate immune response and both are involved in activating the complement system and defending against bacterial pathogens [31-34]. The results of the present study showed that both pcIL-8 and pcENO increased the level of serum lysozyme activity and ACH_50_ activity compared with PBS and pcDNA3.1 , which induced the innate immune responses and activated the protective mechanisms, resulting in higher level of lysozyme activity and ACH50 activity appearing in fish vaccinated with pcENO+pcIL-8. It is speculated that co-injection of pcIL-8 could stimulate the NF-kB/IL-8 signaling pathway in response to inflammation, and the released NF-kB translocates into the nucleus where it activates the transcription or function of downstream immune-related molecules like lysozyme and complement components [35, 36], which together with cytokines and chemokines are the important humoral components for the innate immune system [31]. The difference of these two indexes was that the highest lysozyme activity located in pcENO+pcIL-8 group at 21 days p.v. while the highest ACH50 activity was also detected in pcENO+pcIL-8 group but at 14 days p.v.

Specific antibody induced by subunit or DNA vaccines had been proven to be the basic mechanism of immunoprotection against *S. iniae* infection and been observed in various fish models [25, 26, 37-39]. In this study, pcENO and pcENO+pcIL-8 significantly stimulated the production of anti-rENO specific antibody which were detected at 7 days p.v. and maintained for 56 days p.v. compared with PBS, pcDNA3.1 and pcIL-8 group. Moreover, the antibody level of fish vaccinated with pcENO+pcIL-8 was remarkably higher than that of pcENO from 7 to 56 days p.v. with the peak of antibody titers both appearing at 28 days p.v., which indicated that pcIL-8 functioned for increasing the antibody level induced by pcENO vaccination. However, the time of antibody production and the peak in our study was different or even brought forward comparing with other vaccines against the same pathogen [38-41], chiefly because of the different vaccines and fish species, as well as different immune schedules.

Despite specific antibody is important for the immune response against *S. iniae* infection, it is not sufficient for complete protection which involves both humoral and cellular immunity [42]. Many immune-related molecules or genes related to pro-inflammatory response, humoral immunity and cellular immunity are also contribute to the immune protection. Therefore, transcriptional analysis of immune-related genes including IL-1β, TNF-α, CXCL10, MHC Iα, MHC IIβ, CD4-L2, CD8α and IFN-γ were conducted by qRT-PCR in our study. The results showed that compared with PBS and pcDNA3.1, pcENO and pcIL-8 increased the expression of all investigated genes especially MHC Iα, MHC IIβ, CD8α in pcENO group and IL-1β, TNF-α, CXCL10 in pcIL-8 group, which was consistent with the observation that co-administration of pIL8+ with pMCV1.4-G activated the cytokine response and increased the expression of two pro-inflammatory cytokines (IL-1β and TNF-α) in head kidney of rainbow trout [43]. Moreove, basing on this upregulation, pcENO+pcIL-8 notably induced the expression of all investigated genes compared with pcENO alone (Figure 8). These results shown above suggested that not only pcENO could induce a systemic immune response, but also pcIL-8 could activate inflammatory response and strengthen the immune response in channel catfish against *S. iniae* infection.

RPS as a measure to evaluate vaccine efficacy against pathogen infection, is one of the most visual indices to assess the immuneprotection effect in challenge test [42]. In the present study, pcENO induced a moderate immune protection (with RPS of 60%) for channel catfish against S.in*iae* infection in the challenge experiment of 4 weeks p.v., which was slightly higher than the RPS (50%) of rENO subunit vaccine in our previous study [21] and lower than the RPS (100%) of rENO in zebrafish against same pathogen [39], mainly due to different vaccine types and vaccine delivery route, as well as different fish species and infection dosages. As expected, co-vaccination with pcIL-8 enhanced the protection efficacy of pcENO, resulting in the RPS of 80% in pcENO+pcIL-8 group, which was higher but not significant comparing with the RPS (60%) of pcENO alone by log-rank test at the same time. However, pcENO and pcENO+pcIL-8 could still provide the RPS of 53.33% and 73.33% respectively at the challenge of at 8 weeks p.v. , which were comparable with other DNA vaccines such as pSia10 with RPS of 73.9% in turbot [38] and pSagF with RPS of 78% in Japanese flounder [25] against S.in*iae* at 4 weeks p.v. Moreover, the RPS in group pcENO+pcIL-8 and pcENO showed significant difference (*p* < 0.05).

Taken together, these results indicated that pcIL-8 served as molecular adjuvant co-injection with DNA vaccine not only improve the immune protection but also maintain long period of immunity for channel catfish against S.in*iae* infection. Our study signified that IL-8 hold promise to serve as a potential adjuvant in DNA vaccines against bacterial infections in fish.

## MATERIALS AND METHODS

### Ethics statement

All animal experiments were reviewed and approved by the Committee of the Ethics on Animal Care and Experiments at Sichuan Agricultural University. All experimental procedures were perfomed in accordance with the Guidelines for Experimental Animals maintained by Chinese Ministry of Science and Technology.

### Bacterial strains, plasmids, reagents and growth conditions

S.in*iae* DGX07 is a pathogenic isolate from diseased channel catfish in China and stored at our laboratory, it was cultured in Brain-Heart Infusion (BHI) medium at 37°C. *E. coli* DH5α were used as the host strains for cloning which were routinely grown in Luria-Bertani (LB) medium containing 100μg/ml of ampicillin at 37°C. The plasmids pMD19-T simple (Takara, Japan) and pcDNA3.1 (+) (Invitrogen, USA) were used for T-A cloning and eukaryotic expression, respectively. The recombinant DH5α containing pcDNA3.1-ENO (pcENO) plasmid and recombinant protein rENO were constructed and stored at our laboratory [44].

### Primers design, amplification and cloning of IL-8

In order to enhance the transcription and translation efficiency of IL-8 gene in eukaryotic expression, a Kozak consensus sequence (GCCACCATGG) [45] was added at the 5’ end of the front primers. Besides, a 6× GTG sequence (reverse complement of 6× histidine tag) was also insert at the 5’ end of the reverse primers for the detection of IL-8 expression *in vivo*. The primers were designed based on the channel catfish IL-8 sequence published in GenBank (KP701473) and named as IL-8F (5-CCC AAGCTTGCCACCATGGAGGCTGCAACTCTCACA -3, underlined shown as Hind III site, bold shown as Kozak consensus sequence) and IL-8R1 (5-GGAATTCTCA GTGGTGGTGGTTTTGCTGTTTGATCTTCT-3, underlined shown as EcoR I site, bold shown as reverse complement of 6× histidine tag). The primers IL-8F and IL-8R2 (5-GTGGTGGTGGTGGTGGTGGT-3) were used for the detection of target plasmid DNA *in vivo*.

Total RNA was extracted from head kidney of healthy channel catfish with RNAiso Plus Kit (TaKaRa, Dalian, China) and was reverse-transcribed into first-strand cDNA using PrimeScrip t™ RT reagent Kit with gDNA Eraser (Perfect Real Time) (TaKaRa) according to the manufacturer's instructions. The target gene IL-8 was amplified by PCR with the cDNA template and primers IL-8F/IL-8R under the following conditions: 1 cycle of 95°C for 5min, 30 cycles of 95°C for 1min, 57.6 °C for 30s, and 72°C for 30 s, followed by a final extension of 72°C for 10 min. Then the PCR products were identified on 1% agarose gels, purified using the Agarose Gel DNA Extraction Kit (TaKaRa) and cloned into the pMD19-T vector, followed by transformation into *E. coli* DH5α. The positive recombinant clone was selected using an Amp/IPTG/X-Gal agar plate. The plasmid was extracted from the positive recombinant clone using SanPrep Column Plasmid Mini-Preps Kit (Sangon Biotech, Shanghai, China), identified by PCR under the aforementioned conditions, digested with restriction enzymes Hind III and EcoR I, and fractionated on 1% agarose gels. DNA sequencing was conducted by Sangon Biotech and the correct recombinant plasmid was named as T-IL-8.

### Plasmid construction and preparation

The plasmid T-IL-8 was digested with Hind III and EcoR I and the IL-8-containing fragment was retrieved and inserted into Hind III /EcoR I-digested pcDNA3.1 (+) vector with T4 Ligase (TAKARA), resulting in recombinant plasmid pcDNA3.1/IL-8 (pcIL-8). The plasmid pcIL-8 was also transformed into *E. coli* DH5α, identified and sequenced as above described. Endotoxin-free plasmid DNA was extracted using GoldHi EndoFree Plasmid Maxi Kit (Kangwei, Beijing, China) according to the manufacturer's instructions. The plasmid DNA (pcDNA3.1, pcIL-8 and pcENO) concentration and purity were determined by Nanodrop 2000 (Thermo Scientific, Wilmington, DE, USA) with measuring absorbance at A_260_ and A_260_ /A_280_.

### Preparation of fish and vaccines

Channel catfish (50.0g ±5.0 g) were purchased from a fish farm in Chengdu (Sichuan, China) and acclimatized in the laboratory for 2 weeks before experimental manipulation. The fish were fed a commercial diet daily, and the water was partly replaced every day, maintaining a temperature of 28°C±1°C. Before experiments, fish were randomly sampled from blood, liver, kidney, and spleen, the examination of bacterial recovery indicated no bacteria could be detected and agglutination test showed no reaction between the serum and* S. iniae* DGX07. Fish were anaesthetized with tricaine methanesulfonate (MS222) (Sigma, Beijing, China) prior to injections and blood collection. The plasmid pcDNA3.1, pcIL-8 and pcENO were diluted in PBS to 250 μg/ml, to obtain pcENO+pcIL-8, the plasmid pcENO was mixed with an equal volumes of pcIL-8.

### Vaccination and bacterial challenge

Five hundred channel catfish were divided randomly into five groups (100 fish /group) and injected intramuscularly (i.m.) with 100 μl of PBS (control), pcDNA3.1, pcIL-8, pcENO and pcENO+pcIL-8. At 4 weeks and 8 weeks post vaccination (p.v.). 30 fish from each group were randomly selected and challenged by intraperitoneal injection (i.p.) with 0.2ml of the *S. iniae* strain DGX07 that resuspended in PBS to 6 ×10^7^ CFU/ml [46]. Mortality was monitored over a period of 14 days after the challenge, and dying fish were randomly selected for the examination of bacterial recovery from liver, kidney, and spleen. Relative percent of survival (RPS) was calculated according to the following formula: RPS = [1− (% mortality of vaccinated fish/% mortality of control fish)] × 100 [47]. Serum samples of five fish in each group were collected for assessment of immune related indexes at 1, 3, 7, 14, 21, 28, 42 and 56 days p.v., and head kidney of five fish were taken for qRT-PCR at 24 h post-challenge of 4 week p.v. All vaccination trials were repeated once.

### PCR detection of plasmid DNA in vaccinated fish

Muscle tissues around the injection site of five fish in each group were taken at 7, 14, 28 and 56 days p.v. DNA was extracted from the tissues with MiniBEST Universal Genomic DNA Extraction Kit (TAKARA). PCR was conducted using specific primers (IL-8F/IL-8R2) to plasmid DNA.

### Reverse transcriptase-PCR (RT-PCR) analysis of expression of DNA vaccines

Total RNA was extracted from muscle tissues around the injection site of five fish in each vaccinated group at 14 days p.v. using RNAiso Plus Kit (TaKaRa) and was reverse-transcribed into first-strand cDNA as described above. About 360 bp product of exogenous IL-8 gene segment was amplified with the specific primers IL-8F/IL-8R2 and from the cDNA by RT-PCR using β-actin (ACTB) as an internal control, as well as the amplification of the ENO gene (~1320bp) with the specific primers as described previously [44]. The RT-PCR products were electrophoresed on a 1% agarose gel.

### Immunohistochemical analysis of exogenous IL-8 expression *in vivo*

Immunohistochemistry was performed according to the method of Seo et al. [48] with some modification. Briefly, muscle tissues were taken from five vaccinated fish in each group at 14, 28 and 56 days p.v. The tissue samples were fixed with 4% paraformaldehyde for 48h, followed by dehydrating in ethanol and embedding in paraffin. The sections (5 μm) were cut with a Leica RM2135 microtome (Leica Microsystems, Germany), and incubating with rabbit anti-6-histidine monoclonal antibody (Sangon Biotech, Shanghai, China) and then with goat-anti-rabbit IgG (H+L)-HRP (Sigma, St. Louis, MO, USA). After staining with DAB (Sigma) for 5 to 15 min, the sections were observed by light microscopy (Nikon, Tokyo, Japan).

### Detection of innate immune parameters

Serum lysozyme activity was measured by the turbidimetric method as described previously [21]. Briefly, 150 μL Micrococcus lysodeikticus with a concentration of 0.2 mg/mL (in 0.04 M PBS, pH = 6.2) was added to 15 μL sera in a 96-well U-bottom microtiter plate, quickly mixed by vortex, and the OD520 (UV absorption value at 520 nm) (A_0_) was assayed at 0.5 and 4.5 min after reaction, respectively. Each test was conducted in triplicate. One activity unit of lysozyme (U) was defined as the amount of serum lysozyme that caused a decrease in absorbancy of 0.001 per min at 520 nm.

Alternative complement activity (ACH50) was determined based on haemolysis of rabbit red blood cells (RaRBC) as described by Sunyer and Tort [49]. The volume of serum yielding 50% haemolysis (ACH50) was determined and used to calculate the complement activity of the samples (value of ACH50 is in units per ml)

### Enzyme-linked immunosorbent assay (ELISA)

Specific anti-rENO antibody were determined by ELISA as described previously [21]. Briefly, the rENO was diluted to a concentration of 50μg/mL in a carbonate buffer (pH = 9.6). Each well of the 96-well plate was covered with 100μL diluted rENO overnight at 4°C followed by washing with PBST (0.1% Tween-20 in PBS) and blocking with 3% BSA in PBST for 2 h at 37°C. Serial 2-fold dilutions of sera were added to the wells in triplicate and subsequently incubated for 2 h at 37°C. Rabbit anti-channel catfish IgM antibody (prepared in our laboratory) (1:2000) and goat-anti-rabbit IgG (H+L)-HRP (1:2000) were used as the secondary and tertiary antibodies, respectively. Color development was performed with the TMB kit (Tiangen, Beijing, China). The plates were read at 450 nm with a microplate reader (Bio-Rad, Hercules, USA).

### qRT-PCR analysis of the expression of immune-related genes

Head kidney samples were taken from the fish (five in each group) at 24h post-challenge of 4 weeks p.v. Total RNA extraction and cDNA synthesis were carried out as described above. qRT-PCR was performed with the SYBR^®^ Premix Ex Taq™ II (Tli RNaseH Plus) (TaKaRa) in an ABI StepOnePlus™ System (Applied Biosystems, USA) as described previously [50]. Each assay was performed in triplicate with β-actin (ACTB) as an internal control. The primers used to amplify the immune-related genes have been reported and shown in our previous research [21]. All data are given in terms of relative mRNA.

### Statistical analysis

All statistical analyses were performed with SPSS 19.0 software (SPSS Inc., USA). Mortality data from the challenge experiments were analyzed by the Kaplan-Meier methods and differences among groups were tested using log-rank test. The difference significance of other data were determined using a one-way analysis of variance (ANOVA). In all cases, the significance level was defined as *p* < 0.05 and the results were presented as means± SD (standard deviation).
